# Supplementary material for: Role of ferroelectric polarization during growth of highly strained ferroelectric materials
Source: Nat Commun. 2020 May 26;11:2630. doi: 10.1038/s41467-020-16356-9 (PMC7251112; doi:10.1038/s41467-020-16356-9)
Supplement: Supplementary file 1 — Supplementary Information [file 41467_2020_16356_MOESM1_ESM.pdf]

SUPPLEMENTARY INFORMATION FOR "ROLE OF FERROELECTRIC POLARIZATION DURING GROWTH OF HIGHLY STRAINED FERROELECTRIC MATERIALS" BY RUI LIU ET AL.

A. Structural characterization using laboratory-based X-ray diffraction.

Crystal structures of the films were determined by X-Ray Diffraction on a Bruker D8-Discover high-resolution X-Ray Diffractometer. The wavelength used was 1.5406 Å. Here we show three $BaTiO_3$ films grown on top of 3 unit cell $PbTiO_3$ ultra-thin films as examples. The growth temperatures are below, around or above the ferroelectric transition temperature of 3 unit cell $PbTiO_3$ ultrathin film (550° C). All samples showed epitaxial growth and the out of plane lattice parameters are close to each other (See Supplementary Fig. 1).

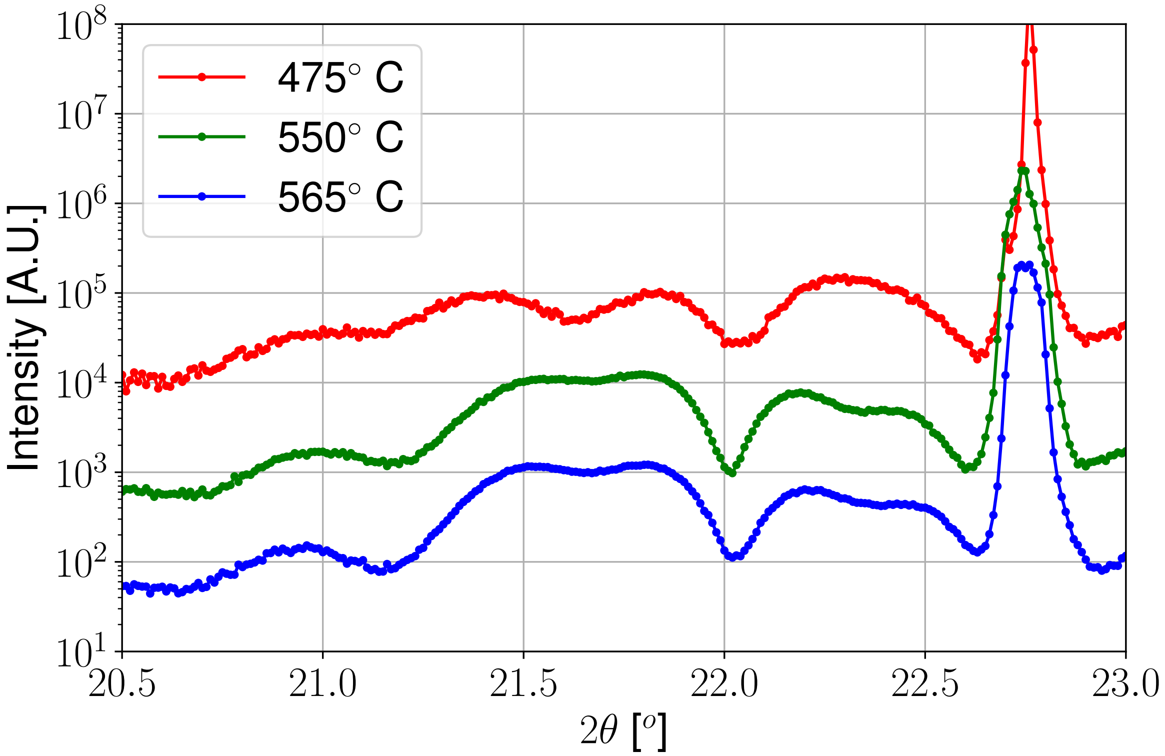

Supplementary Fig 1. Examples of $\theta - 2\theta$ scans around (0 0 1) Bragg peak: $BaTiO_3$ films grown on 3 unit cell $PbTiO_3$ films at growth temperatures of 475° C (red), 550° C (green), 565° C (blue). The growth temperatures are below (red), around (green) or above (blue) the ferroelectric transition temperature of 3 unit cell $PbTiO_3$ film. Source data are provided as a Source Data file.

B. Topography characterization.

The topography of films was characterized using an atomic force microscope (MFP-3D, Asylum Research). The surfaces of all the films were clean and atomically flat with single unit cell steps of 0.4 nm (See Supplementary Fig. 2).

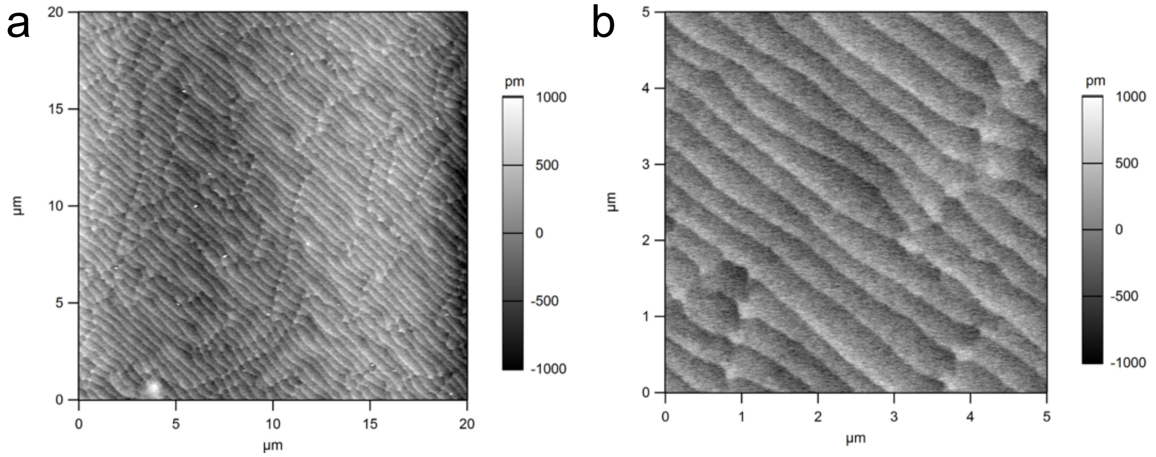

Supplementary Fig 2. Examples of topography of $BaTiO_3$ films: AFM topography of $BaTiO_3$ film grown on 3 unit cell of $PbTiO_3$ films with scan sizes (20 × 20) μm^2 and (5 × 5) μm^2.

C. Ferroelectric polarization measurements using PUND.

Ferroelectric polarization was measured using a PUND technique. PUND, or Positive Up Negative Down, is a pulse train that uses repeating up and then repeating down pulses to first switch the polarization and then gather information about non-switching currents from the second pulse. Using this technique any non-switching contributions to the current were subtracted out. Polarizations of three $BaTiO_3$ films grown on 3 unit cell of $PbTiO_3$ films at different temperatures were presented in Supplementary Fig. 3, inserted with switching currents collected during the PUND measurements. Multiple measurements were conducted for each sample to obtain accurate results with statistical significance.

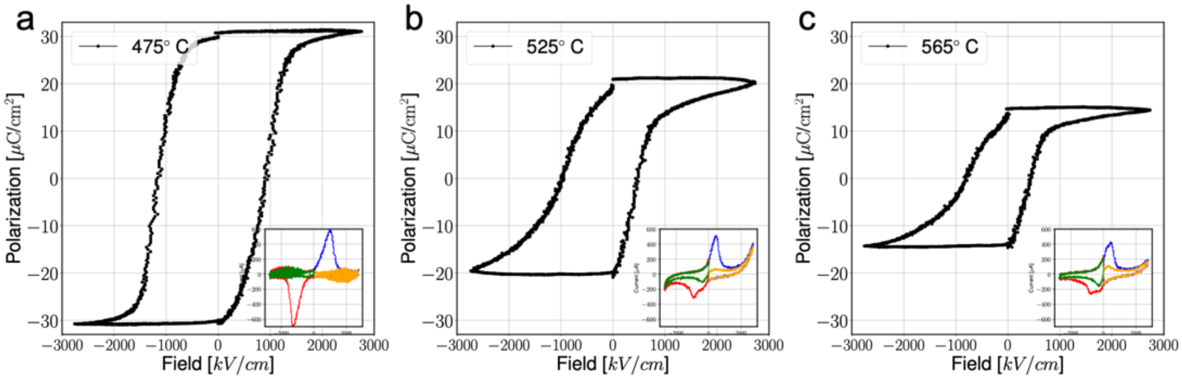

Supplementary Fig 3. Examples of ferroelectric hysteresis loops measured using a PUND technique: Ferroelectric hysteresis loops of $BaTiO_3$ films grown on 3 unit cell of $PbTiO_3$ films at growth temperatures of 475° C (a), 525° C (b), 565° C (c). The inserted figure at the right bottom corner of each figure was the switching currents collected in the PUND measurement versus the applied electrical field. Color guide: P pulse (blue), U pulse (orange), N pulse (red), D pulse (green). Source data are provided as a Source Data file.

D. Electrical properties of the films.

To measure the piezoelectric response of the samples DART (Dual Amplitude Resonance Tracking) Piezo-Force Microscopy (PFM) measurements were made using Co/Cr-coated AFM tip as the top electrode. The measured amplitudes and phases response recorded during the measurement is shown in Supplementary Fig. 4 (a,b). It can be seen that all the samples are very good ferroelectrics at room temperature and they have similar piezoelectric coefficients d_{33}. The dielectric constant was also measured as a function of applied voltage using an LCR circuit and all samples displayed the expected butterfly loop characteristic of strongly ferroelectric materials (See Supplementary Fig. 4 (c)). All the films have similar dielectric constants between 150 to 200.

One very intriguing observation we have made is that while the magnitude of piezoelectric coefficients is similar for all of the films, those films were grown on ferroelectric $PbTiO_3$ substrates have an electromechanical resonance frequency about 10 % lower compared to those grown on paraelectric substrates (See Supplementary Fig. 5). The ferroelectric polarization and the surface domain configuration can affect the elastic properties of the samples, which

in turn had an influence on the electromechanical resonance frequency.

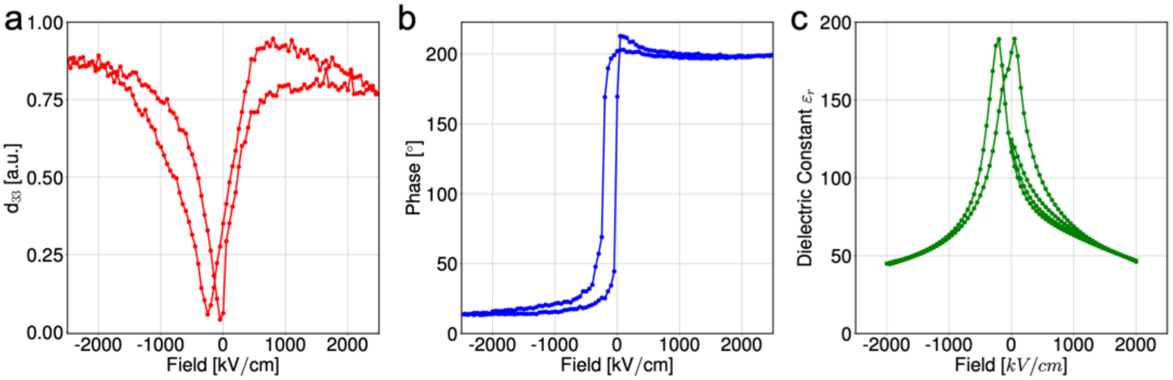

Supplementary Fig 4. Electrical properties of the films: (a,b) The measured amplitudes and phases (a and b, respectively) of the tip response during the DART PFM measurements were plotted against the applied field. (c) The dielectric constant was also measured as a function of applied field. The butterfly loop characteristic showed strong ferroelectricity. Source data are provided as a Source Data file.

E. Growth monitor using the anti-Bragg peak.

To monitor the growth and also to calibrate the growth rates, the intensity of the reflected signal at the $(0\,0\,\frac{1}{2})$ Bragg position (to minimize bulk Bragg diffraction) was measured as a function of growth time. The oscillations of the reflectivity signal provide a measurement of surface roughness similar to reflection high energy electron diffraction (RHEED). While the RHEED technique is not appropriate here due to the presence of magnetic fields and does not provide the same amount of structural information as x-ray diffraction can. The maxima intensity correspond to the completed layers while the low signals correspond to incomplete layers. The oscillations indicate that the BTO grown on paraelectric substrate did not maintain a smooth surface, while BTO grown on ferroelectric PTO continues to grow smoothly in a layer-by-layer mode for many layers (See Supplementary Fig. 6). The ferroelectric polarization of PTO underneath helps maintains the BTO growth in a smooth layer-by-layer mode.

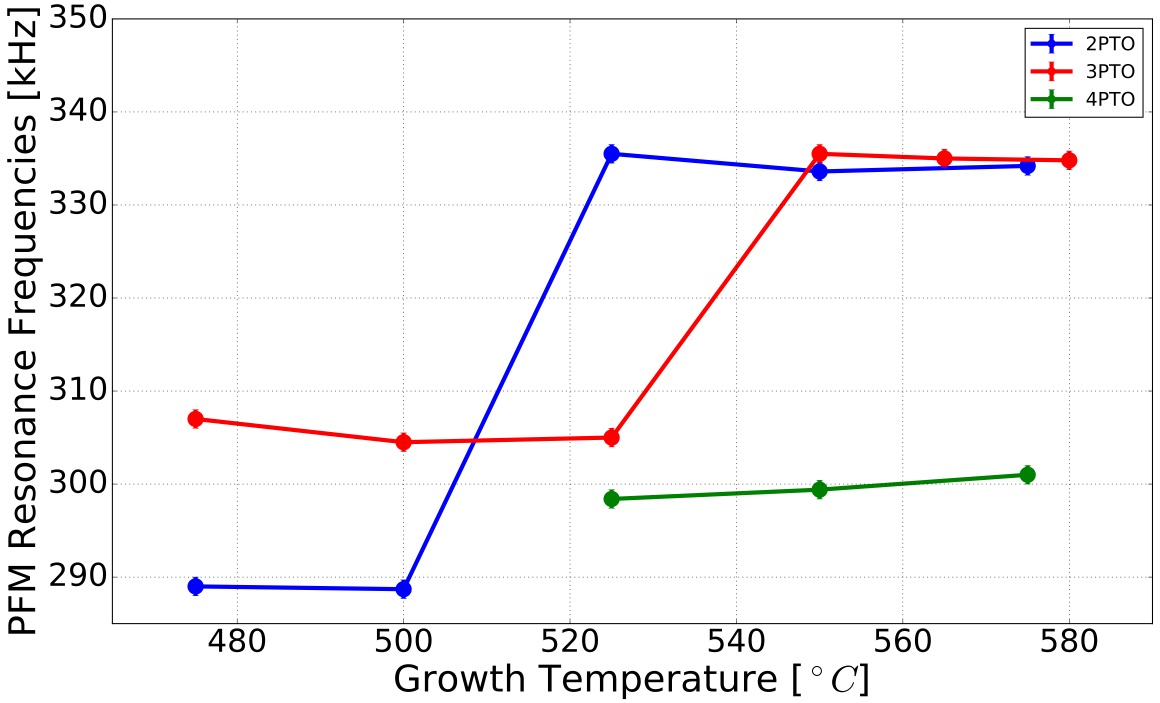

Supplementary Fig 5. Electromechanical resonance frequences of samples measured by DART PFM: Electromechanical resonance frequences of $BaTiO_3$ films grown on 2 (blue), 3 (red), 4 (green) unit cell $PbTiO_3$ films versus growth temperatures. Source data are provided as a Source Data file.

F. In-situ X-ray scans near (1 0 1) peak.

The evolution of the relaxation of BTO can be obtained from the scans near the (1 0 1) peak (Two extreme examples were presented in the paper). All the films grow strained with the substrate at the beginning of growth, then the lattice parameters a and c start to merge at different thickness and tend toward bulk lattice parameters in the end. (See Supplementary Fig. 7 (a,b)). The area of the relaxed BTO was calculated by adding up all pixels above a certain limit in the relaxed region (See Supplementary Fig. 7 (c,d)).

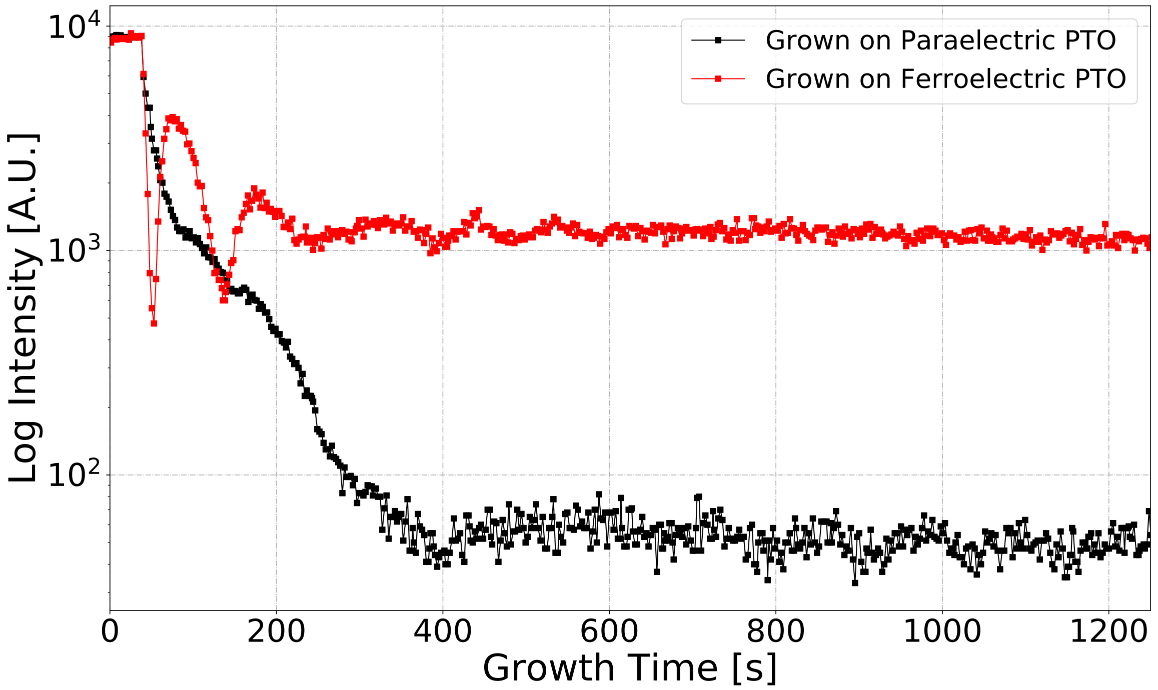

Supplementary Fig 6. Growth monitor using the anti-Bragg peak: Intensity of the $(0\,0\,\frac{1}{2})$ anti-Bragg peak versus growth time for $BaTiO_3$ films grown on paraelectric (black) and ferroelectric (red) $PbTiO_3$ films. It can be seen that the BTO grown on paraelectric PTO did not grow in a smooth way, while BTO grown on ferroelectric PTO grows smoothly in the first few layers.

G. Reciprocal space growth movies obtained by in-situ x-ray scans around (1 0 1) peak.

The reciprocal space maps can be assembled into continuous movies that allow the observation of the growth process (Supplementary Movie 1). Two series of samples were grown: one keeps the thickness of PTO films to be the same while varying the growth temperatures, and another keeps the growth temperatures to be the same while varying the thickness of PTO films. The polarization of PTO substrates was manipulated by both ways (see the inserted image on top). Epitaxially strained growth was observed during the whole process, while the BTO films start partially relaxing at different growth time. In general, the ferroelectric polarization underneath can help constrain BTO films to the lattice parameters of the substrates, maintaining large compressive strain in the film. The relaxation process was

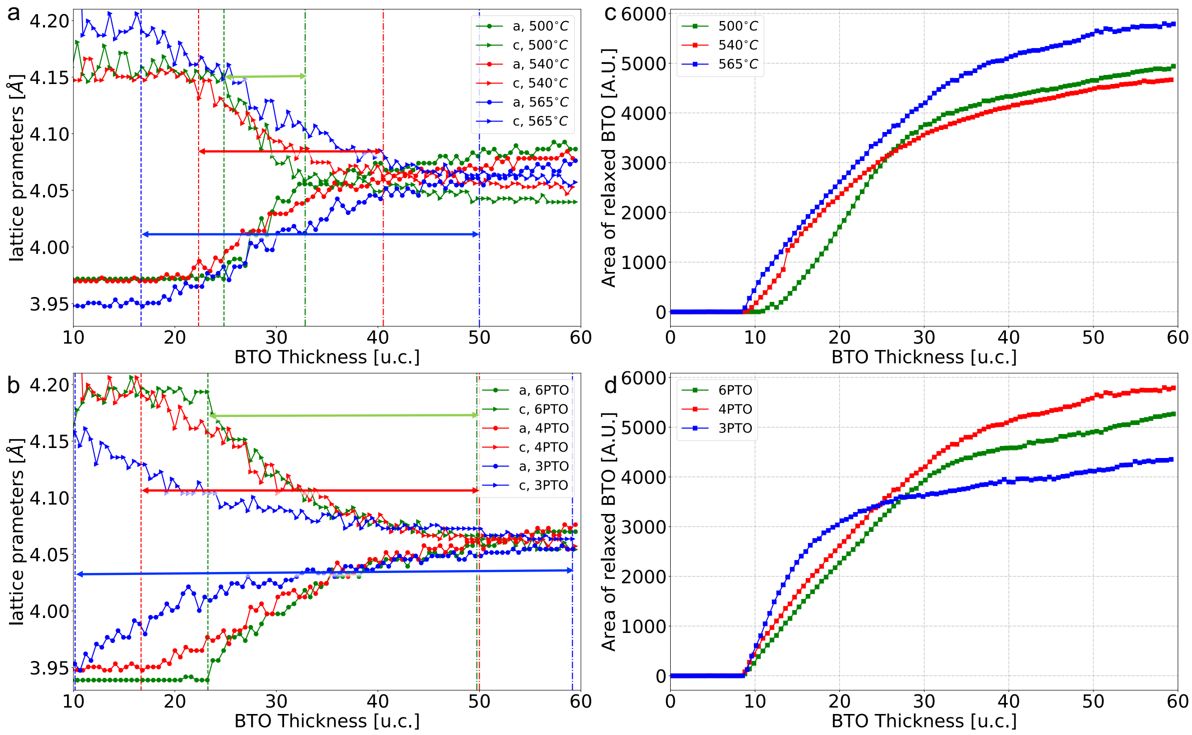

Supplementary Fig 7. Evolution of lattice parameters and sizes of relaxed parts of BTO films obtained by in-situ x-ray scans near (1 0 1) peak: (a,b) In-plane lattice parameters a (circle date point markers) and out of plane lattice parameters c (triangle data point markers) of relaxed BTO were plotted against BTO thickness for two series of samples: one keeps the unit cell layers of PTO films to be 4 while varying the growth temperatures (a, green: 500° C, red: 540° C, blue: 565° C), and another keeps the growth temperatures to be 565° C while varying unit cell layers of PTO films (b, green: 6 layers, red: 4 layers, blue: 3 layers). The vertical dashed line indicates the beginning of relaxation and the vertical dash-dotted line indicates the end of relaxation for each film. The horizontal arrow points out the whole range of the relaxation process. (c,d) Area of relaxed part of BTO films plotting against BTO thickness for two series of samples: one keeps the unit cell layers of PTO films to be 4 while varying the growth temperatures (a, green: 500° C, red: 540° C, blue: 565° C), and another keeps the growth temperature to be 565° C while varying unit cell layers of PTO films (b, green: 6 layers, red: 4 layers, blue: 3 layers). Source data are provided as a Source Data file.

delayed by the polarization of the substrates and the relaxation mode was also changed.

H. Analysis of domains from in-situ x-ray data around (0 0 1) peak.

The evolution of domains during the growth can be obtained from in-situ x-ray scans around (0 0 1) peak. First, we integrated the intensity along the Q_z direction around the Bragg peak of BTO. Then we fitted the Bragg peak plus two domain peaks using three independent Lorentz functions along the Q_x direction (See Supplementary Fig. 8). Finally, we calculated the domain size from the distances between the Bragg peak and the Domain peaks. Several fitting methods were used to cross-check the results. After applying the method to every scan of all films, we obtained the evolution of domain sizes for all samples.

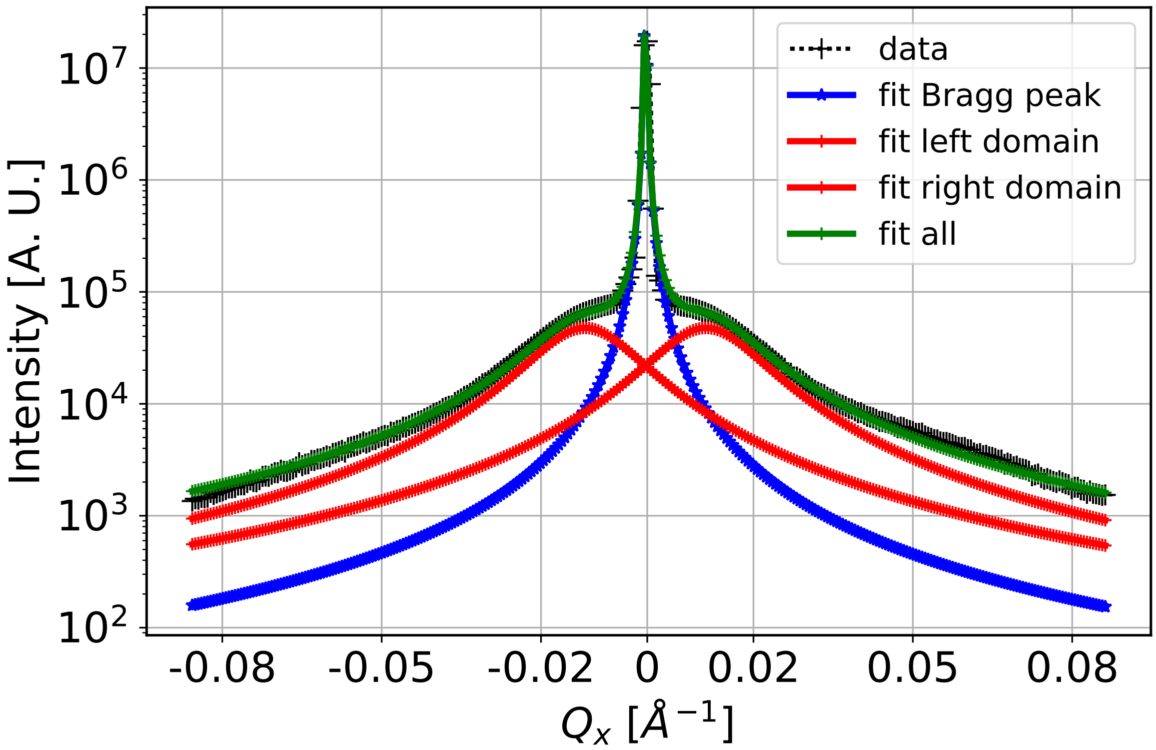

Supplementary Fig 8. An example of domain fitting: The integrated plot around the Bragg peak of BTO along the Qx line together with a fit (green) to the data (black). The Bragg peak (blue) and the domain diffuse (red) were fitted using three independent Lorentz functions.

I. Reciprocal space growth movies obtained by in-situ x-ray scans around (0 0 1) peak.

The reciprocal space growth movies around (0 0 1) peak (Supplementary Movie 2) were obtained using the same method as (1 0 1) growth movies. The polarization of PTO substrates was also inserted in the movie.

J. Grazing-incidence in-situ x-ray scans near (1 0 0) peak.

Further insight into the difference in the in-plane relaxation process can be obtained by performing grazing-incidence X-ray scattering around the (1 0 0) peak. Examples from the beginning to the end of growth of BTO film grown on ferroelectric and paraelectric PTO substrates are shown in Supplementary Fig. 9 and Supplementary Fig. 10. Two types of relaxation process were observed: Process 1 (around the green dashed line in Supplementary Fig. 9 (c,f)) with larger Q_z and Process 2 (around the blue dashed line in Supplementary Fig. 9 (c,f)) with smaller Q_z. These two processes happened at different Q_x positions at the beginning of growth and merged to the same Q_x positions at the end of the growth if grown on the ferroelectric substrate (Supplementary Fig. 9). By contrast in the samples grown on paraelectric substrates the two processes are at the same Q_x positions during the whole growth process (Supplementary Fig. 10).

K. Response of domain scattering to applied electric field

In subsequent experiments at the CHX beamline at NSLS-II, the scattering around the (0 0 2) peak for selected samples from the growth experiments at ISR were measured under applied electric field. In Supplementary Fig. 11 we show examples of the scattering at 5 different points of sinusoidal voltage waveform that was applied to a Pd electrode on the sample labelled as 9u.c. 560°C in Fig. 4.

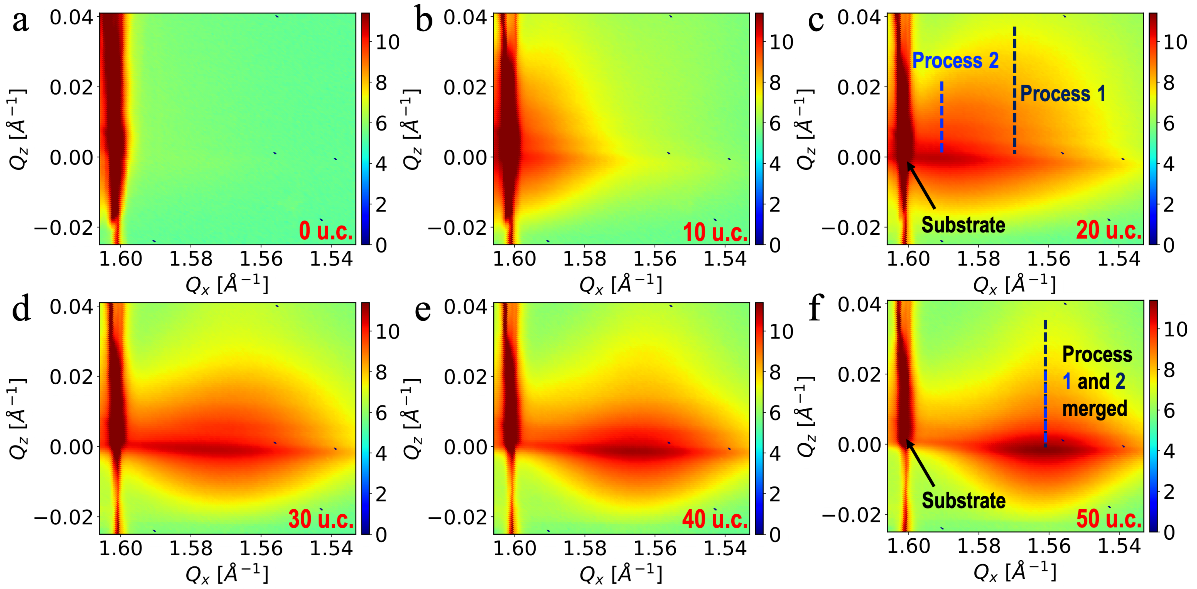

Supplementary Fig 9. Examples of reciprocal space maps obtained by in-situ Grazing-incidence x-ray scans around (1 0 0) peak for BTO films grown on ferroelectric substrate after (a) 0 u.c., (b) 10 u.c., (c) 20 u.c., (d) 30 u.c., (e) 40 u.c., and (f) 50 u.c. BTO films were grown. Two types of relaxation processes were seen which begin at different Q_x positions at the beginning of growth and merge to the same Qx positions at the end of the growth.

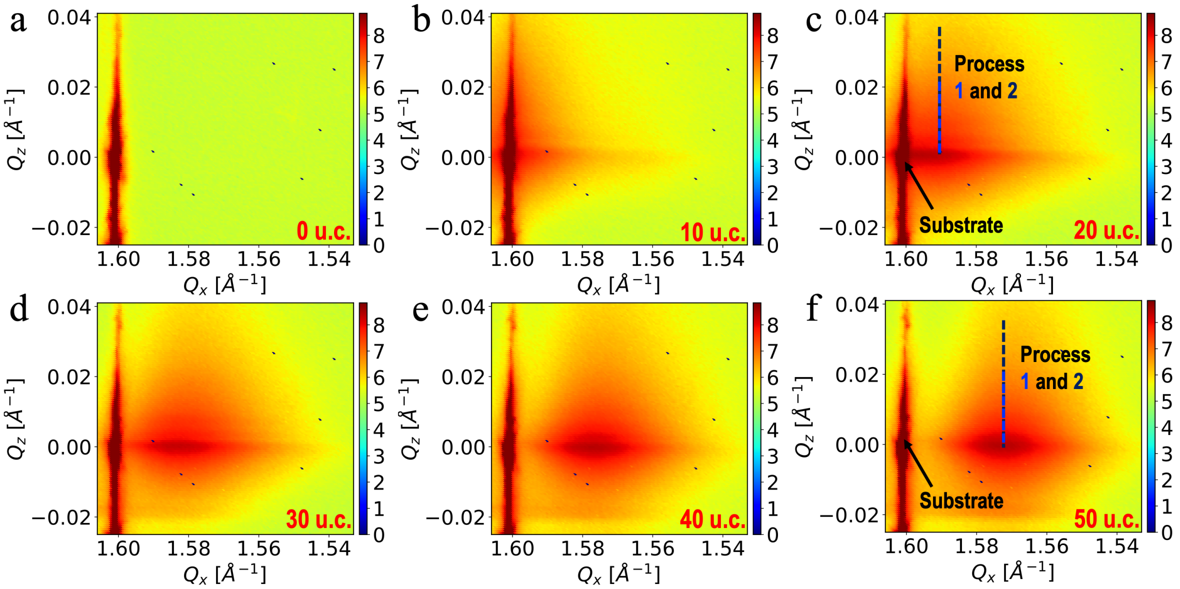

Supplementary Fig 10. Examples of reciprocal space maps obtained by in-situ Grazing-incidence x-ray scans around (1 0 0) peak for BTO films grown on paraelectric substrate after (a) 0 u.c., (b) 10 u.c., (c) 20 u.c., (d) 30 u.c., (e) 40 u.c., and (f) 50 u.c. BTO films were grown. The two processes are at the same Q_x positions during the whole growth process.

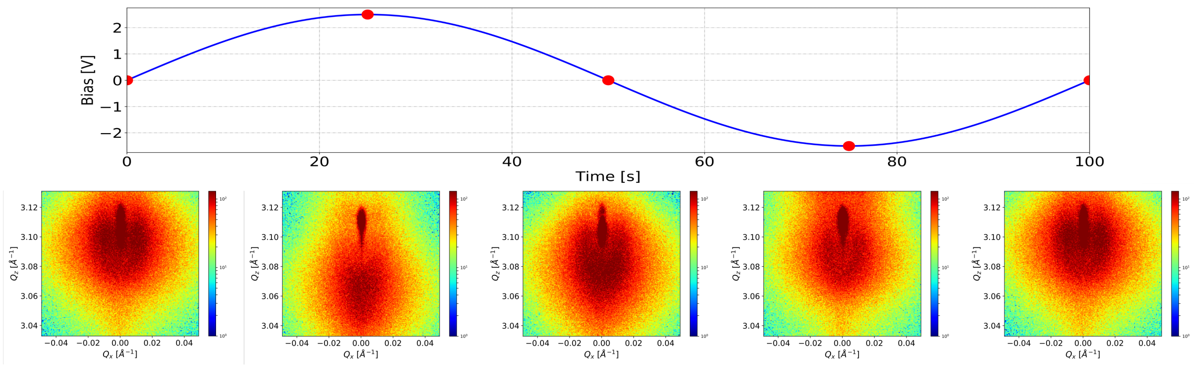

Supplementary Fig 11. Motion of the scattering from domains around the (0 0 2) BTO peak of the BTO film grown on 9 u.c. thick PTO at ISR under applied electric field. This data was taken at the CHX beamline at NSLS-II at room temperature post-deposition. The field was applied via a Pd top electrode.
