# Supplementary material for: Role of ferroelectric polarization during growth of highly strained ferroelectric materials
Source: Nat Commun. 2020 May 26;11:2630. doi: 10.1038/s41467-020-16356-9 (PMC7251112; doi:10.1038/s41467-020-16356-9)
Supplement: Supplementary file 2 — Description of Additional Supplementary Files [file 41467_2020_16356_MOESM2_ESM.pdf]

Description of Additional Supplementary Files

File name: Supplementary movie 1
Description: Reciprocal space maps around (101) that have been assembled into continuous movies that allow the observation of the growth process. The panels in the movie correspond to different samples matching the arrangement in Fig. 5 of the paper.

File name: Supplementary movie 2
Description: Reciprocal space maps around (001) that have been assembled into continuous movies that allow the observation of the growth process. The panels in the movie correspond to different samples matching the arrangement in Fig. 4 of the paper.
